# Tailoring neurosurgical operating room education to medical undergraduates: Integrative review and meta-synthesis

**DOI:** 10.1016/j.bas.2024.104131

**Published:** 2024-11-04

**Authors:** Aaron Lawson McLean, Tui Lin Yen, Felipe Gutiérrez Pineda

**Affiliations:** a Department of Neurosurgery, Jena University Hospital – Friedrich Schiller University Jena, Jena, Germany; b Department of Education, China Medical University Hospital, Taichung, Taiwan; c School of Medicine, College of Medicine, China Medical University, Taichung, Taiwan; d Department of Neurosurgery, School of Medicine, University of Antioquia, Medellin, Colombia

**Keywords:** Medical students, Neurosurgical education, Operating room, Patient safety, Teaching strategies

## Abstract

**Introduction:**

Integrating medical students into the neurosurgical operating room (OR) presents significant pedagogical challenges, compounded by the phenomenon of neurophobia, or aversion to neuroscience. Despite the importance of early neurosurgical exposure, there is a lack of structured educational strategies for undergraduates.

Research Question How can neurosurgical OR education be effectively tailored to address undergraduate medical students' educational needs and mitigate neurophobia?

**Material and methods:**

This study employs an integrative approach, combining narrative synthesis and critical interpretive synthesis (CIS). A comprehensive literature review was conducted across databases such as PubMed, Scopus, and ERIC. Key themes and patterns were identified through iterative reading, coding, and thematic analysis. CIS was utilized to integrate these themes into a coherent theoretical framework, focusing on applicability, feasibility, and educational impact.

**Results:**

The study presents twelve targeted strategies to optimize neurosurgical OR education. These include preoperative planning, fostering a positive learning environment, emphasizing technological integration, involving students in decision-making, prioritizing safety, providing regular feedback, facilitating active participation, leveraging teachable moments, managing time constraints, offering follow-up opportunities, emphasizing professionalism, and fostering a learning culture.

**Discussion and conclusion:**

This framework addresses a critical gap in neurosurgical education for undergraduates, tackling neurophobia and enhancing learning experiences. By integrating educational theories with practical insights, it offers a robust, adaptable approach suitable for various global resource settings. Through continuous evaluation and refinement, these strategies can evolve to meet the dynamic demands of neurosurgical education, preparing students to navigate the complexities of modern neurosurgical practice with confidence and competence.

## Highlights

• Addresses neurophobia in medical students through targeted pedagogical strategies.
• Comprehensive framework for neurosurgical OR education for undergraduates.
• Integrates narrative and critical interpretive synthesis methodologies.
• Emphasizes active student participation and decision-making in the OR.
• Proposes adaptable strategies for varied global educational resource settings.

## 1 Introduction

The initiation of medical students into the intricate world of neurosurgical practice, particularly within the confines of the operating room (OR), represents a pedagogical challenge of considerable complexity. This complexity is not only due to the inherent technical demands and the high-stakes nature of neurosurgery but also because of the unique educational needs and experiences of undergraduate medical trainees. The neurosurgical OR is a milieu where meticulous skill, profound knowledge, and critical decision-making converge, necessitating an educational approach that is both comprehensive and nuanced (Croghan et al., 2019; O'Neill et al., 2018; Ravi et al., 2022; Duy et al., 2019).

A significant obstacle in this educational endeavor is the phenomenon of “neurophobia” – a term coined to describe the apprehension and aversion that many medical students feel towards the neurosciences. This aversion often stems from the perceived complexity of neuroanatomy and neurophysiological concepts, as well as the high stakes involved in neurosurgical procedures (Lukas et al., 2014; Ridsdale et al., 2007; Solorzano and Józefowicz, 2015). The prevalence of neurophobia has been well-documented in medical education literature and is a cause for concern, not only because it can influence career choices away from neurology and neurosurgery but also because it may compromise the competence of future physicians in managing neurological conditions (Pakpoor et al., 2014; Knight et al., 2017).

Furthermore, the neurosurgical OR presents specific educational challenges. These include the limited margin for error, the necessity of understanding complex anatomical structures, and the need to make swift yet precise decisions (Ekhator and Rak, 2022). These factors demand heightened attention from both educators and students, posing a significant challenge to traditional pedagogical methods. The dynamic roles that medical students can play in the OR – ranging from observers to active assistants – further complicate the establishment of a standardized educational approach (O'Neill et al., 2018; Marwan et al., 2012).

Despite these challenges, literature on structured educational strategies for undergraduate medical students in neurosurgical OR settings is sparse. Existing frameworks primarily serve residents or postgraduate trainees, with undergraduate medical education receiving less focus. This oversight indicates a significant gap in medical education: the absence of a well-defined set of strategies to enhance the learning experiences of medical students in the demanding neurosurgical environment.

This paper addresses this gap by proposing a comprehensive framework of educational strategies specific to medical students on neurosurgical rotations. These strategies, rooted in medical education theory, are tailored to the unique challenges and learning opportunities presented by the neurosurgical OR. The aim is to assist educators in equipping medical students with an in-depth understanding of neurosurgery, while upholding the highest standards of patient care. By tackling the issue of neurophobia and catering to the specific educational needs of medical students in neurosurgery, this paper contributes to the ongoing efforts to improve medical education in high-stakes surgical settings.

While the primary focus of this manuscript is on undergraduate medical students, many of the educational strategies proposed here are also applicable to postgraduate training programs, particularly in their early stages. By optimizing neurosurgical education for medical students, these strategies lay the groundwork for more advanced learning during residency and beyond.

## 2 Methods

This study utilized an integrative approach combining narrative synthesis and critical interpretive synthesis (CIS) to develop a targeted educational framework for neurosurgical OR teaching aimed at undergraduate medical students (Depraetere et al., 2021; Dixon-Woods et al., 2006). A comprehensive literature search was conducted across major academic databases, including PubMed, Scopus, and ERIC, covering the period from January 2000 to August 2023. The search strategy focused on articles discussing neurosurgical OR education, pedagogical approaches for medical students, and relevant teaching frameworks.

In the initial phase, narrative synthesis was employed to extract and distill key themes and patterns from the literature. This iterative process involved multiple rounds of reading, coding, and thematic analysis. Subsequently, the CIS methodology was applied to critically interpret the relationships between these themes and integrate them into a coherent theoretical framework. This process was guided by principles of reflexivity, with attention paid to the authors’ dual roles as clinicians and educators.

The CIS approach enabled the synthesis of a wide range of evidence types, from empirical studies to reflective practice insights, and emphasized applicability and feasibility in diverse educational settings. Ethical considerations were central to the framework, particularly in balancing educational opportunities with patient safety.

Further details on the CIS process, including the systematic literature review, coding procedures, and specific analytical methods, are provided in Appendix A. Information regarding the search strategy can be found in Appendix B.

## 3 Results

**Fig. 1:**
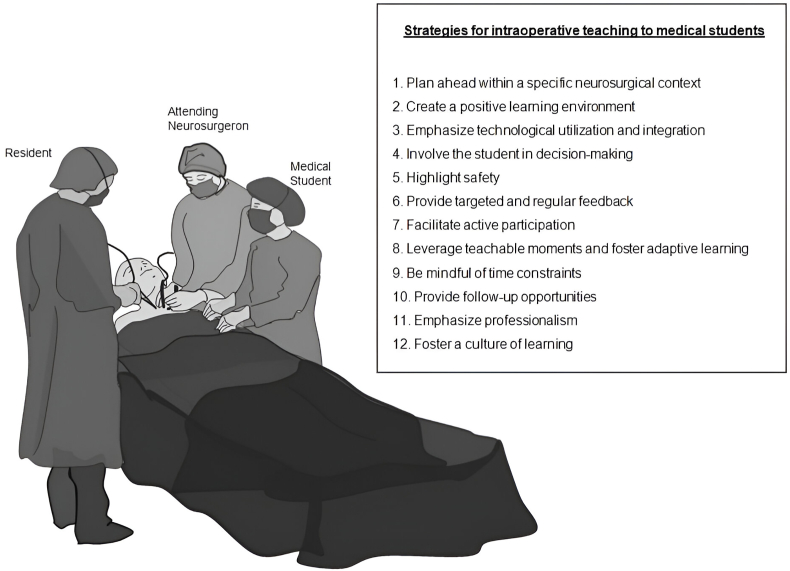
Framework of twelve pedagogical strategies for enhancing neurosurgical operating room education tailored to undergraduate medical students. Developed through integrative review and meta-synthesis, these strategies encompass preoperative planning, student involvement, technology integration, feedback, and professionalism, addressing the unique pedagogical challenges in neurosurgical education.

In the ensuing results section, we delineate a compendium of twelve targeted strategies designed to optimize the educational milieu within the neurosurgical operating room (Fig. 1). These strategies, refined through an integrative methodological framework, aim to reconcile medical educational theory, empirical insights from relevant literature, and experiential wisdom gleaned from scholarly consultations and extensive clinical practice. While the emphasis is on the unique pedagogical challenges of the neurosurgical environment, the applicability of these guidelines may transcend specialty boundaries. It is essential to bear in mind that these strategies are presented as part of an ongoing discourse and should be tailored to specific contexts, learners, and resources available.

### 3.1 Plan ahead within a specific neurosurgical context

In line with the educational theory of “backward design,” setting clear, specific learning objectives pertinent to the neurosurgical procedure in question is of paramount importance for teaching in the neurosurgical OR (Robertson and Bradley, 2017). This approach involves defining the desired learning outcomes first and then devising a teaching plan to achieve these objectives.

For instance, in the context of a scheduled microvascular decompression, the learning objectives might encompass an understanding of the relevant cranial anatomy, the rationale behind the chosen surgical approach, and mastering the nuances of utilizing the surgical microscope. Such targeted learning objectives provide a roadmap for the teaching plan and ensure that the teaching aligns with the actual surgical procedure.

The inherent unpredictability of the OR schedule due to emergencies or alterations in patient status may pose an obstacle in planning ahead. However, this challenge can be mitigated by preparing several versatile teaching plans tailored to different common procedures. This approach facilitates adaptability while preserving an organized, goal-oriented teaching approach.

Furthermore, learning objectives should ideally be communicated with students ahead of the procedure, allowing them to prepare accordingly and align their self-study with the upcoming surgical case. This proactive engagement not only maximizes the educational value of the OR experience but also fosters an active learning environment conducive to deeper understanding and long-term retention.

### 3.2 Create a positive learning environment

Cognitive load theory suggests that the cognitive processing capacity of learners is significantly influenced by the environmental context in which learning takes place (Venkat et al., 2020). This theory underscores the importance of establishing a learning environment that is supportive and non-threatening to effectively facilitate the comprehension and assimilation of intricate neurosurgical concepts. The nature of the neurosurgical OR, with its sophisticated technology and life-critical procedures, can often present as an intimidating environment for medical students (Ismail et al., 2023; Zoli et al., 2022; Woelfel et al., 2023). This perception can inadvertently elevate the learners' cognitive load, hindering their ability to process new information and skills.

To overcome this barrier, it's crucial to provide medical students with an in-depth orientation to the OR. This should include basic familiarization with unique neurosurgical technologies, such as navigations station and surgical microscopes, along with their safe and effective operation. It should also encompass the roles and responsibilities of each team member, highlighting the collaborative nature of patient care in neurosurgery.

Further, fostering a culture of open communication and mutual respect, where students feel safe to ask questions or seek clarification, is instrumental in minimizing cognitive load. Regular debriefing sessions after the procedure can also help students to reflect on their experience, consolidate their learning, and alleviate any residual anxiety. This multi-faceted approach creates an environment conducive to learning, effectively harnessing the OR's potential as an invaluable educational platform for medical students and budding neurosurgeons.

### 3.3 Emphasize technological utilization and integration

Neurosurgery, with its unique intersection of medical acumen and technology, offers immense learning opportunities for medical students. Applying multimedia learning theory, educators can use digital imaging, imaging and navigation platforms and other technological resources to augment OR teaching (Grech, 2018; Yue et al., 2013). Technologies such as digital imaging, intraoperative videos, and advanced surgical tools with high-definition monitors such as the Zeiss Kinevo 900 microscope with integrated exoscope and endoscope (Zeiss Qevo tool) and the Olympus ORBEYE 4K-3D exoscope can be harnessed to supplement students' understanding of intricate neuroanatomy and nuanced surgical techniques (Belykh et al., 2018; Layard et al., 2022). These technologies, with features such as 3D visual options, provide immersive experiences, offering students a more comprehensive insight into the surgical process, especially in situations where traditional microscope usage might limit the student's view.

However, it is critical to note that these technological resources may not be universally available across all OR settings, particularly in regions with limited access to high-end equipment. In low-resource environments, alternative strategies may include using smartphone-adapted endoscopic views or basic video recording equipment for case review and debriefing. The utilization of simpler, cost-effective technological alternatives ensures that the educational objectives are not compromised while accounting for economic and resource limitations.

The challenges in technological integration extend beyond mere availability and may include a steep learning curve, especially for those unfamiliar with these advanced tools. A structured approach to overcoming this involves breaking down the operation of each device into its constituent steps, allowing for gradual increases in complexity as the student demonstrates growing competence. Additionally, financial constraints and the susceptibility to technical malfunctions should not be underestimated as barriers to technological integration.

Ultimately, the judicious and safe application of technology should be underscored. Students must be educated to understand that while technology can greatly assist, it does not supplant the need for clinical acumen and adherence to established surgical principles. Thoughtful incorporation of technology, adapted to the specific context of each OR environment, enables not only the enhancement of learning experiences but also fosters technological literacy, a requisite skill set in contemporary neurosurgical practice (Korn et al., 2022; Wang et al., 2022).

### 3.4 Involve the student in decision-making

Constructivist learning theory posits that learners are not merely passive receivers of information, but active participants in the knowledge construction process (Thomas et al., 2014). Drawing from this theoretical background, it is beneficial to involve medical students in the neurosurgical operating room in a manner that engages them in the complexities of patient care, without compromising safety or integrity of care. This should not be misconstrued as entrusting critical decisions to students, which would indeed be unsafe and inappropriate. Rather, this involves facilitating opportunities for students to observe and understand the decision-making process in neurosurgery.

Encourage the students to think through clinical problems, articulate their thought processes, and predict what they anticipate the attending neurosurgeon might do next. This fosters critical thinking and augments their understanding of clinical decision-making. One potential challenge is that students may hesitate to express their thoughts in the high-stakes environment of the neurosurgical OR. Therefore, it is essential to create a psychologically safe space where students feel comfortable discussing their reasoning. A strong emphasis should be placed on the fact that these discussions are purely for educational purposes and all final clinical decisions rest solely with the attending neurosurgeon.

### 3.5 Highlight safety

Emphasizing safety is paramount in any medical discipline, but it takes on amplified significance within the high-stakes arena of neurosurgery. Building upon the situated learning theory, which posits that learning is most efficacious in authentic contexts, teaching safety principles should occur directly within the operative environment (O'Brien and Battista, 2020; Shinkaruk et al., 2023; Zickuhr et al., 2021). In neurosurgery, this entails not merely maintaining sterility while manipulating complex tools such as microscopes or endoscopes but also understanding the judicious use of neuromonitoring instruments to safeguard patient wellbeing. This multi-layered approach imbues learners with a comprehensive understanding of safety measures, ranging from infection control to neurological function preservation.

A key challenge encountered in safety education is transforming safety procedures from an abstract notion to a concrete practice, a shift that often occurs only after witnessing or experiencing a safety breach (Alsulami et al., 2022; Patey et al., 2007; Runciman et al., 2009). To pre-empt such situations, educators can instill robust safety consciousness by routinely demonstrating and discussing safety protocols. Real-time teaching of critical incidents management, including complications, can help cement these safety principles.

Further, integrating surgical safety checklists into the teaching curriculum, emphasizing effective communication within the team, and encouraging students to voice their safety concerns can promote a safety culture in the neurosurgical OR. By infusing safety into every aspect of the OR teaching, we can ensure that our students are well-prepared to navigate the challenging terrain of modern medicine while keeping patient safety at the forefront.

### 3.6 Provide targeted and regular feedback

Feedback in the neurosurgical operating room plays a pivotal role in the learning process for medical students. This principle is grounded in Kolb's experiential learning theory, which posits that effective learning occurs when individuals are given the opportunity to reflect on their experiences, absorb constructive feedback, and apply it in subsequent scenarios (Almalag et al., 2022; Yoshimura et al., 2020). This process is particularly pertinent in the neurosurgical OR, where the real-time application of learned skills and knowledge can greatly enhance student learning outcomes.

The challenge in the OR environment, however, is providing timely, continuous feedback that maintains a positive and conducive learning atmosphere. Potential obstacles include the time-intensive nature of neurosurgical procedures and the inherent risk of dampening the confidence of the learner with overly harsh criticism. To address these issues, consider adopting a modified version of the “feedback sandwich” technique, often referred to as the “Pendleton's rules” method (Burgess et al., 2020; Pendleton et al., 2003). This technique begins with the learner first identifying what went well, followed by the teacher's affirmation of these points. Next, the learner identifies areas they feel could have been improved, and lastly, the teacher provides further insight and advice on these areas.

Not only does this model create a constructive dialogue, but it also empowers the learner to self-evaluate before receiving external feedback, fostering an environment of self-directed learning. Furthermore, it ensures that feedback is both specific and immediately applicable, thereby aiding in the consolidation of the student's learning experiences. Lastly, it alleviates some of the time pressures associated with the traditional feedback model, as students begin the reflection process independently. Through this approach, medical students in the neurosurgical OR can maximize their learning while preserving a positive educational experience.

### 3.7 Facilitate active participation

The concept of active learning, wherein students are actively engaged in their educational process rather than passively receiving information, is a cornerstone of effective education. This principle holds significant relevance in the highly technical and hands-on field of neurosurgery, where the tangible integration of knowledge and skills can enhance the depth and breadth of learning. However, the practical application of active learning theory in the neurosurgical OR necessitates a delicate balance between student involvement and optimal patient care.

Active participation in the neurosurgical OR can take numerous forms. For the uninitiated student, this might begin with simpler tasks such as retracting or suctioning under supervision, advancing to more complex tasks like navigating an endoscope or aiding in the interpretation of intraoperative neurophysiological monitoring data. The challenge lies in ensuring that these experiences augment the student's learning without compromising patient safety or procedural efficiency.

An approach to overcome this challenge is the concept of “graded responsibility,” a practice in which students are entrusted with tasks proportionate to their skill level and understanding, with the complexity of these tasks increasing as they demonstrate proficiency (Gheihman et al., 2021; Lai et al., 2020; Schnapp et al., 2019). This principle facilitates a systematic progression of students' skill acquisition, allowing them to gain confidence and competence in a safe, controlled manner. In parallel, educators should constantly assess and respond to students' learning needs and progress, thereby ensuring an individualized learning experience. The use of effective feedback mechanisms, as discussed in the previous point, plays a pivotal role here. Thus, through active participation coupled with a system of graded responsibility, the neurosurgical OR can become a fertile ground for effective and impactful medical student learning.

### 3.8 Leverage teachable moments and foster adaptive learning

Teachable moments are spontaneous events that can offer significant learning experiences, often stimulating profound cognitive shifts. These moments align closely with the Transformative learning theory, which posits that impactful learning can incite changes in learners' perspectives or comprehension (Jones, 2010; Tims, 2014). In the neurosurgical OR, these teachable moments could arise from unexpected findings, complications, or even exceptionally well-executed procedures. However, the unpredictable nature of such moments, coupled with the highly demanding environment of neurosurgery, present distinct challenges. The key concern is ensuring patient safety while capitalizing on these educational opportunities. Addressing this involves a twofold approach.

First, educators must exhibit agility in teaching – the ability to swiftly shift from their planned instructional course to leverage a sudden learning opportunity (Frenk et al., 2022; Bashir et al., 2021; Carey and Kies, 2022). This could mean pausing during a procedure to explain an unexpected finding, or a post-procedure debriefing to discuss how a complication was managed. However, the discussion should never compromise patient safety or procedural efficiency, and thus, the timing and extent of these explanations need to be judiciously determined.

Second, educators should instill in students the value of adaptability and resilience in learning – the capacity to learn from unexpected situations and adapt their understanding accordingly (Babenko and Lee, 2022; Lawson, 2023). This might prepare them for a career in neurosurgery, where unexpected findings and complications are part and parcel of the specialty. Therefore, by acknowledging the inherent unpredictability of the neurosurgical OR and skillfully integrating it into the teaching process, educators can maximize teachable moments, thereby fostering an environment conducive to transformative learning.

### 3.9 Be mindful of time constraints

In the neurosurgical OR, time is a precious commodity. Neurosurgical procedures can be intricate and lengthy, often making it challenging to allocate sufficient time for comprehensive teaching without compromising patient care. Therefore, employing a time-efficient teaching strategy, such as the just-in-time teaching (JiTT) concept, becomes critical in such environments. JiTT is an instructional strategy designed to ensure that teaching aligns with the learner's needs at the exact moment they arise (Dominguez et al., 2018; Schuller et al., 2015). It capitalizes on the teachable moments that spontaneously occur during complex procedures.

For example, when a particular anatomical landmark becomes visible during the operation or a certain technical maneuver is performed, the attending neurosurgeon can immediately seize the opportunity to explain the relevance and the associated neurosurgical knowledge to the student.

By practicing JiTT, educators can provide efficient, targeted teaching that dovetails seamlessly with the surgical procedure. This strategic approach facilitates the reinforcement of theory with practice, enhancing understanding and retention, and optimally utilizing the limited teaching time in the OR.

### 3.10 Provide follow-up opportunities

Constructivist learning theory posits that learning is an ongoing process, with each experience building on the last (Chuang, 2021). In the context of neurosurgical OR education, this learning model highlights the importance of post-operative discussions and reviews as essential components in solidifying and contextualizing the knowledge and skills gained in the OR.

The nature of post-operative learning opportunities extends beyond the mere discussion of surgical outcomes. They provide a platform for reviewing the surgical procedure in retrospect, discussing intraoperative decisions and actions, clarifying doubts, reviewing pathological findings, understanding post-operative care, and even preparing for potential complications. By revisiting the surgical experience in a less time-pressured environment, students can enhance their understanding of clinical decision-making, patient management, and technical skills.

To illustrate, after a complex brain tumor resection, the attending neurosurgeon might discuss the specifics of tumor anatomy, deliberate on decision-making regarding the extent of resection, review intraoperative images or videos, and analyze the histopathological report. Such a session not only reinforces the operative learning but also broadens the students' perspective on patient care.

Despite the inherent value, integrating such post-operative educational discussions can be challenging due to the demanding schedules of both the educators and learners. To navigate this challenge, educators could adopt a systemic approach by scheduling regular debriefing sessions, perhaps aligning them with existing academic activities or conferences. Leveraging digital tools for communication can also facilitate these discussions, overcoming constraints of time and location. Further, incorporating these educational encounters as a recognized element of the curriculum can ensure their regular occurrence.

In sum, capitalizing on the opportunities for post-operative education and integrating them into the standard neurosurgical teaching protocol enriches the learning experience, aligns with the constructivist learning theory, and ensures a more comprehensive and enduring learning outcome.

### 3.11 Emphasize professionalism

The concept of the “hidden curriculum” in medical education underscores the significant impact that non-explicit teaching and observational learning have on shaping a medical student's professional development (Lawrence et al., 2018; Lempp and Seale, 2004). This concept becomes acutely important within the OR environment, where students not only absorb procedural knowledge and technical skills but also assimilate attitudes, ethical standards, and patterns of professional behavior exhibited by their superiors.

Professionalism in the OR encompasses an array of attributes, including respect for all team members, adherence to patient confidentiality, prioritizing patient care, and maintaining a high standard of ethical conduct. For instance, a surgeon's diligent cross-checking of the patient's identity and surgical site, their interaction with the nursing staff, their response to intraoperative complications, or their handling of sensitive patient information are all observed and internalized by the student. These moments of observed professionalism contribute significantly to shaping the student's future professional identity.

However, fostering professionalism in the OR comes with inherent challenges. The high-stress environment, demanding surgical procedures, and long working hours could potentially lead to occasional lapses in professional conduct. To counter this, it is essential for surgical educators to consciously role-model professional behavior and create a culture that values and promotes professionalism. Explicit discussions around expectations of professional conduct, reflection on observed behaviors, and immediate, constructive feedback on any lapses can further reinforce this learning.

For example, debriefing sessions could be used to discuss observed professional behaviors or to address any identified lapses. If an inappropriate comment was made during a stressful moment in surgery, this could be addressed in the debriefing, with the involved individual explaining their understanding of the lapse and suggesting how they could manage such situations differently in the future.

In essence, demonstrating professionalism in the neurosurgical OR is not just a passive expectation but an active, integral component of medical education. When approached with intent, it enhances the overall educational experience, aligning with the principles of the hidden curriculum and significantly influencing the professional development of medical students.

### 3.12 Foster a culture of learning

Vygotsky's social learning theory posits that learning is intrinsically a social process, with knowledge being co-constructed within a community of learners (Vygotsky et al., 1978). This concept holds significant relevance to teaching in the OR, where a dynamic team of individuals, each with unique skills and insights, work together. The cultivation of a learning culture within this setting, where every team member - from attending surgeon to medical student - is both a learner and a teacher, creates a vibrant, engaging, and inclusive learning environment.

In the neurosurgical OR, this learning culture can manifest itself in various ways. For example, a neurosurgical resident might explain the rationale behind a surgical step to a medical student, or a scrub nurse might share their insights about instrument handling. An attending neurosurgeon might learn a new perspective from a question posed by a junior team member. In this way, the OR becomes a dynamic space of shared learning and knowledge exchange, enriching the learning experience for all involved.

However, the establishment and nurturing of this culture can be hindered by several factors. The hierarchical nature of medical education, combined with the high-stakes, high-pressure environment of neurosurgery, may create barriers to open communication and mutual learning. Team members may be hesitant to ask questions or provide feedback due to fear of seeming inadequate or disrupting the established authority dynamics.To overcome these challenges, it is crucial to actively encourage a culture of curiosity, open communication, and mutual respect. This can be accomplished by promoting an environment where all team members feel comfortable sharing their knowledge, asking questions, and providing constructive feedback. Role modeling this behavior by senior members, praising instances of mutual learning, and addressing any barriers to open communication promptly and constructively can further help in fostering this culture.

Therefore, by embracing the principles of social learning, the OR can be transformed into a rich, interactive learning environment. This approach aligns with the evolving trends in medical education towards more collaborative, learner-centered models and ultimately helps train more competent, confident, and curious potential future neurosurgeons (Beckman and Lee, 2009; Martin et al., 2021; Vinson et al., 2022; Yang, 2023).

## 4 Discussion

The results of this study underscore the complexity and multifaceted nature of educating medical students in the neurosurgical operating room. The proposed strategies integrate a range of educational theories and practical insights, reflecting the intricacy of neurosurgical pedagogy. Key themes emerge, including the variability in available educational resources and the limitations imposed by these constraints.

An important consideration in enhancing neurosurgical education is promoting neurosurgery not only as a technical discipline but also as a craft and fulfilling professional endeavor (Alnaami et al., 2022). Highlighting the blend of manual dexterity, intellectual challenge, and critical decision-making that defines neurosurgery may play a role in attracting students to the specialty. By emphasizing these aspects, educators could inspire more students to view neurosurgery as both a viable and rewarding career path.

### 4.1 Global variability and resource constraints

One of the principal challenges in the global implementation of neurosurgical educational strategies lies in the profound variability of resources and infrastructure across different regions. The disparity between high-income countries, with their access to cutting-edge technology and extensive educational resources, and low- and middle-income countries, where resources are often limited, cannot be overstated (Liang et al., 2016). This disparity not only influences the availability of advanced technological aids but also impacts the fundamental structure of educational programs, leading to significant inconsistencies in the quality and uniformity of neurosurgical training worldwide (Sarpong et al., 2022).

In addressing this challenge, it is prudent to recognize that the operationalization of these educational strategies is contingent upon the resources available, which can vary dramatically. The presence of advanced technologies, such as 3D visualization or sophisticated endoscopy tools, commonly found in high-resource settings, should not be seen as a prerequisite for effective neurosurgical teaching. Instead, the core principles underpinning these recommendations must be adaptable and scalable, allowing for contextual customization based on the resource availability in each setting. For instance, in environments where high-end imaging modalities are not readily available, the focus could shift towards enhancing fundamental skills such as anatomical identification, tissue handling, and the proficient use of basic surgical instruments. This approach ensures that the essence of neurosurgical education – imparting vital skills and knowledge – remains intact, irrespective of the technological landscape.

The dual approach required to mitigate these challenges involves, firstly, the development of context-specific strategies tailored to resource-constrained environments. In settings where sophisticated equipment is scarce, leveraging more affordable technology and emphasizing traditional mentorship models can provide a partial compensation for the absence of high-end resources. Secondly, there is a pressing need for enhanced international collaboration and resource-sharing initiatives (Almeida et al., 2018). Such efforts could include exchange programs, online training modules, and partnerships between institutions from varying economic backgrounds. These collaborative efforts would not only facilitate the sharing of knowledge and resources but also promote a more egalitarian approach to neurosurgical education, ultimately striving towards a more uniform standard of training across diverse global regions.

This integrative approach underscores the necessity of a flexible, context-sensitive framework in neurosurgical education, one that can accommodate the vast spectrum of global resource variability while maintaining the integrity and effectiveness of the educational experience.

### 4.2 Addressing gender-specific challenges

Additionally, it is essential to address the unique challenges faced by female medical students, who now comprise the majority of those entering medical school (Boyle, 2024). Pregnancy and family responsibilities are often viewed as barriers to pursuing surgical careers, particularly in demanding fields like neurosurgery (Dixon et al., 2019). Recent policies introduced in 2024 by major surgical organizations have aimed to address these concerns. For example, the US American College of Surgeons (ACS) has revised its guidelines to emphasize the importance of workplace support for pregnant surgical trainees, including individualized schedule modifications, protected time for prenatal appointments, and safe accommodations for operative duties (American College of Surgeons, 2024). These initiatives ensure that surgical trainees can continue their professional development without compromising personal choices related to pregnancy and family life.

In Germany, a specific guideline has been introduced for pregnant neurosurgeons by the German Association for Neurosurgery (Deutsche Gesellschaft für Neurochirurgie; DGNC) and the Professional Association of German Neurosurgeons (Berufsverband der Deutschen Neurochirurgie; BDNC), which outlines the conditions under which pregnant neurosurgeons can continue to perform surgeries. This includes allowing participation in elective surgeries with appropriate precautions, adjustments to the physical demands of surgical work, and exclusion from radiation exposure (Conzen-Dilger et al., 2023). These policies reflect a broader effort to balance maternal health and career progression.

Promoting awareness of these supportive measures within undergraduate neurosurgical education is crucial. It helps female medical students understand that pursuing a surgical career, including neurosurgery, is not incompatible with pregnancy and family life (Smith et al., 2024). Early exposure to these initiatives can inspire confidence in female students, ensuring that gender-specific concerns do not deter them from choosing neurosurgery as a viable and rewarding career path, fostering greater gender equity within the field.

### 4.3 Integration of technology in neurosurgical education

The integration of advanced technology in neurosurgical education is a double-edged sword. While it offers unparalleled opportunities for enhancing learning, it also introduces challenges such as the need for specialized training and the potential for over-reliance on technological aids. The balance between embracing technological advancements and maintaining fundamental surgical skills is critical. Educators should ensure that technology complements, rather than replaces, traditional teaching methods and clinical acumen.

### 4.4 Ethical considerations and patient safety

A paramount concern in neurosurgical education is the ethical obligation to ensure patient safety. The involvement of medical students in the neurosurgical OR should never compromise patient care. This ethical dimension necessitates a careful balancing act between providing educational opportunities and maintaining the highest standards of patient safety (Lawson McLean and Lawson McLean, 2024). Educators must be vigilant in supervising students, ensuring that their involvement is always aligned with their level of competence and does not pose any risk to patients.

### 4.5 Adapting to evolving neurosurgical practices

Neurosurgery is a rapidly evolving field, with ongoing advancements in surgical techniques, technology, and patient care protocols. This dynamism presents a challenge for educators to continuously update the curriculum and teaching methods. It is imperative for neurosurgical educators to stay abreast of these changes and incorporate them into their teaching strategies, ensuring that students receive education that is current and relevant.

## 5 Conclusion

In conclusion, it is evident that the current landscape of educational strategies within the neurosurgical OR has been primarily focused on residents. This focus has inadvertently created a pedagogical gap for medical students, a concern that gains additional significance in the context of the documented phenomenon of neurophobia – an excessive apprehension towards the neurosciences prevalent among medical students (Tarolli and Józefowicz, 2018; Jozefowicz, 1994; Shiels et al., 2017). The resolution of this issue necessitates a sophisticated understanding of the principles of medical education, coupled with an appreciation of the specific demands of neurosurgical procedures.

The framework proposed in this paper aims to enhance the educational experience of medical students in the neurosurgical OR. It is grounded in the integration of established educational theories with practical insights derived from neurosurgical practice. This integrated approach is designed to meet the unique educational challenges inherent in neurosurgery. However, the effective implementation of these strategies demands an acute awareness of the variability of resources available globally and a dedication to tailoring these strategies to meet the needs of diverse local contexts.

Moving forward, the trajectory of neurosurgical education should be towards the development of teaching methods that are universally applicable, yet flexible enough to be adapted to a variety of resource environments. It is crucial to continually evaluate and refine these educational strategies, ensuring they remain effective and pertinent in the context of a rapidly evolving medical field.

Moreover, it is imperative to recognize that neurosurgical education, while inherently specialized, holds significant value for medical students who may not necessarily pursue a residency in neurosurgery or neurology. The skills and knowledge imparted in the neurosurgical OR extend beyond technical surgical prowess, encompassing critical thinking, precision, and ethical considerations that are fundamental to all fields of medicine. Thus, neurosurgical education, targeted towards medical students, is not only essential but also relevant. The framework proposed here aims to evolve continuously, equipping future clinicians with the skills and knowledge necessary to excel in the multifaceted and demanding environment of contemporary medical practice.

## Authorship statement

ALM, TLY and FGP have jointly contributed to all stages of this work, encompassing the drafting, critical revising for intellectual content, and final approval of the manuscript for publication. All authors share equal responsibility for the content and integrity of the work.

## Ethical approval

This work has followed appropriate ethical guidelines. Formal ethical approval was not required according to applicable legislation and institutional guidance.

## Data availability

No new data were generated or analyzed in support of this research. The study is based on previously published data, which are cited in the manuscript as references.

## Funding

No specific funding was received for this work.

## Declaration of competing interest

The authors declare that they have no known competing financial interests or personal relationships that could have appeared to influence the work reported in this paper.
