# Supplementary material for: Tailoring neurosurgical operating room education to medical undergraduates: Integrative review and meta-synthesis
Source: Brain Spine. 2024 Nov 4;4:104131. doi: 10.1016/j.bas.2024.104131 (PMC11584684; doi:10.1016/j.bas.2024.104131)
Supplement: Multimedia component 1 [file mmc1.docx]

**Appendix A**

**Comprehensive Methodological Overview**

**1. Conceptual underpinning and methodological orientation**

Our methodological approach is predicated on the integration of narrative synthesis and critical interpretive synthesis (CIS), frameworks often employed in the humanities and qualitative research. This orientation acknowledges the complex, nuanced nature of educational strategies within the neurosurgical OR and leverages a comprehensive, interpretative analysis to construct a meaningful, contextually grounded educational framework [12, 13]. This method is particularly suited to synthesize diverse types of evidence, including theoretical literature, empirical studies, and reflective practice insights, into a cohesive narrative.

**2. Narrative synthesis for educational strategy development**

The narrative synthesis began with a comprehensive literature review, aimed at collating existing knowledge on neurosurgical OR education for medical undergraduates. The inclusion criteria for the literature review were: (1) articles published in peer-reviewed journals, (2) studies focusing on neurosurgical education in the OR setting, (3) publications discussing pedagogical approaches for medical students, and (4) articles in English. Key databases such as PubMed, Scopus, and the Education Resources Information Center (ERIC) were systematically searched. The search strategy included a combination of keywords and phrases related to "neurosurgery", "medical education", "operating room training", and "undergraduate medical curriculum". For detailed search strategies employed across PubMed, Scopus, and ERIC databases, refer to the supplementary file provided (Appendix B). The time frame for the literature search spanned from January 2000 to August 2023, ensuring the inclusion of both seminal and recent studies relevant to the topic. Exclusion criteria encompassed non-peer-reviewed articles, publications not specifically related to neurosurgical OR education, and studies focusing solely on postgraduate or residency training. The search strategy was designed to encompass a wide range of sources, including academic journals, educational theory texts, and grey literature, to ensure a thorough representation of the field. This phase was not limited to systematically reviewed articles but also included opinion pieces, reflective essays, and case studies, recognizing the value of experiential knowledge in educational settings.

Key themes, patterns, and gaps in the literature were identified through an iterative process of reading, coding, and thematic analysis. This process was informed by hermeneutic principles, allowing for a deep engagement with the texts and an appreciation of the nuanced perspectives on neurosurgical education. The synthesis of these narratives enabled the distillation of core educational strategies and principles that are both evidence-based and reflective of the collective wisdom in the field.

**3. CIS for framework integration**

Following the narrative synthesis, a CIS was employed to integrate the identified educational strategies within a coherent theoretical framework. CIS is a methodology that allows for the incorporation of a wide range of evidence types and emphasizes the generation of new theoretical insights through the synthesis process. This phase involved a critical examination of the relationships between identified themes, the exploration of underlying assumptions in the literature, and the integration of educational theories relevant to neurosurgical OR teaching. The CIS process facilitated a reflexive, analytical dialogue between the authors, the literature, and the emergent framework, guided by questions of applicability, feasibility, and educational impact. This dialogic process was instrumental in refining the educational strategies and situating them within a broader conceptual framework that addresses the unique challenges and opportunities of neurosurgical OR education.

**4. Reflexivity and ethical consideration**

A reflexive approach was maintained throughout the research process, acknowledging the authors' positions as both practitioners and researchers in neurosurgery and medical education. This reflexivity informed the interpretation of literature and the integration of strategies, ensuring that the framework remained grounded in practical realities and ethical considerations of teaching in high-stakes environments.

In summary, the methodology employed for this study represents an innovative amalgamation of narrative synthesis and critical interpretive synthesis, tailored to the unique context of neurosurgical OR education for medical undergraduates. By leveraging these methodologies from the humanities, the study crafts a nuanced, deeply contextualized educational framework that resonates with the complexities of neurosurgical teaching and learning. This approach underscores the value of integrating diverse evidence types and reflective practice insights, enriching the academic discourse on medical education in specialized, high-stakes clinical settings.
