# Supplementary material for: Tailoring neurosurgical operating room education to medical undergraduates: Integrative review and meta-synthesis
Source: Brain Spine. 2024 Nov 4;4:104131. doi: 10.1016/j.bas.2024.104131 (PMC11584684; doi:10.1016/j.bas.2024.104131)
Supplement: Multimedia component 2 [file mmc2.docx]

**Appendix B**

**Comprehensive Literature Search Strategy**

This appendix details the search strategy employed to gather literature for the study. It includes the specific databases searched (PubMed, Scopus, ERIC), the combination of keywords and phrases used (e.g., "neurosurgery", "medical education", "operating room training", "undergraduate medical curriculum"), and the inclusion and exclusion criteria applied. The search covered publications from January 2000 to August 2023, encompassing peer-reviewed articles, educational theory texts, and grey literature to ensure a thorough representation of the field.

**PubMed search strategy:**

(neurosurgery OR neurosurgical) AND (medical education OR medical teaching OR clinical education) AND (operating room OR surgical education OR surgical training) AND (undergraduate medical curriculum OR medical students OR medical undergraduates) AND ("2000/01/01"[Date - Publication] : "2023/08/31"[Date - Publication])

**Scopus search strategy:**

TITLE-ABS-KEY((neurosurgery OR neurosurgical)) AND TITLE-ABS-KEY((medical education OR medical teaching OR clinical education)) AND TITLE-ABS-KEY((operating room OR surgical education OR surgical training)) AND TITLE-ABS-KEY((undergraduate medical curriculum OR medical students OR medical undergraduates)) AND PUBYEAR > 1999 AND PUBYEAR < 2024

**ERIC search strategy:**

(neurosurgery OR neurosurgical) AND (medical education OR medical teaching OR clinical education) AND (operating room OR surgical education OR surgical training) AND (undergraduate medical curriculum OR medical students OR medical undergraduates) AND (publication date:2000-2023)
